# ROS Promote Hypoxia-Induced Keratinocyte Epithelial-Mesenchymal Transition by Inducing SOX2 Expression and Subsequent Activation of Wnt/*β*-Catenin

**DOI:** 10.1155/2022/1084006

**Published:** 2022-01-06

**Authors:** Yan Shi, Shang Wang, Ronghua Yang, Zhenmin Wang, Weiwei Zhang, Hongwei Liu, Yuesheng Huang

**Affiliations:** ^1^Department of Wound Repair and Institute of Wound Repair, The Second Clinical Medical College, Jinan University (Shenzhen People's Hospital), Shenzhen 518020, China; ^2^The First Affiliated Hospital, Jinan University, Guangzhou, China; ^3^Chongqing Key Laboratory of Traditional Chinese Medicine for Prevention and Cure of Metabolic Diseases, Chongqing Medical University, Chongqing, China; ^4^Department of Burn Surgery, The First People's Hospital of Foshan, Foshan 528000, China; ^5^Department of Plastic Surgery, The First Affiliated Hospital of Jinan University, Innovative Technology Research Institute of Plastic Surgery, Key Laboratory of Regenerative Medicine, Ministry of Education, Guangzhou, Guangdong Province, China

## Abstract

We previously showed that wound-induced hypoxia is related to keratinocyte migration. The ability of keratinocytes within wound healing to undergo epithelial to mesenchymal transition (EMT) contributes significantly to the acquisition of migratory properties. However, the effect of hypoxia on keratinocyte EMT on wound healing and the potential mechanism are poorly documented. This study first demonstrated that reactive oxygen species (ROS) appear to be an essential signalling mediator in keratinocytes with increased EMT and migration subjected to hypoxic conditions. Next, we showed that the expression of sex-determining region Y-box 2 (SOX2), a stemness-associated molecule, is ROS-dependent under hypoxia and that SOX2 inhibition in keratinocytes dramatically prevented hypoxia-induced EMT and migration. In addition, *β*-catenin was found to be a potential molecular target of SOX2, and the activation of Wnt/*β*-catenin was required for hypoxia-induced EMT and migration. Using an *in vitro* skin culture model and an *in vivo* skin wound model, our study further reinforced the critical role of ROS in inducing EMT through SOX2 expression and subsequent activation of Wnt/*β*-catenin, allowing for rapid reepithelialization of the wound area. Taken together, our findings reveal a previously unknown mechanism by which hypoxia promotes wound healing by promoting reepithelialization through the production of ROS, inducing keratinocyte EMT and migration via the enhancement of SOX2 and activation of Wnt/*β*-catenin.

## 1. Introduction

Mammalian cutaneous wound healing is a well-coordinated process that involves three consecutive but overlapping phases: inflammation, reepithelialization, and maturation. During the wound reepithelialization phase, keratinocytes migrate into the wound site, proliferate, and differentiate to reestablish barrier function and homeostasis of the skin. Dysfunctional keratinocyte migration is an important cause of unsatisfactory wound repair [1, 2]. Remarkably, the movement of keratinocytes during wound healing is thought to involve a process known as epithelial to mesenchymal transition (EMT), in which stationary epithelial cells lose apical-basal polarity and cell-cell adhesion and acquire motility [3]. Due to the key role of EMT in ensuring keratinocyte movement and further reepithelization, a better understanding of the mechanisms underlying wound-induced keratinocyte EMT is very important.

Following acute injury, the epidermis on the wound site is normally deprived of O_2_ owing to the loss of vascular structure and heightened oxygen consumption [4]. Furthermore, low O_2_ tension is a vital component of the epidermal microenvironment that contributes to skin homeostasis and development. Our previous studies have shown that wound-induced hypoxia (2% O_2_) enhances keratinocyte migration [5], but the underlying mechanisms involved in hypoxia-mediated keratinocyte motility have not been fully elucidated. In addition, accumulating studies have revealed that hypoxia exerts positive effects in the regulation of EMT in cancer cells [6]. Since keratinocytes under wound-induced hypoxic conditions undergo EMT with the acquisition of migratory potential during wound healing, we postulate that hypoxia contributes to keratinocyte EMT, thereby facilitating keratinocyte migration. However, the underlying mechanisms are rarely investigated, and further investigation is required.

Hypoxia induces reductive carboxylation to adapt cells to a low oxygen environment, which was shown to increase reactive oxygen species (ROS) generation [7]. Many studies have shown a strong relationship between ROS and hypoxia-mediated cell fate [8, 9]. Our group previously reported that hypoxia-induced ROS facilitated the galvanotaxis of keratinocytes, which was inhibited by scavenging ROS with N-acetyl cysteine (NAC), a well-known ROS scavenger [10]. Additional studies have indicated that the altered production of ROS is responsible for EMT and migration in cancer cells [11]. However, the relationship between ROS and keratinocyte EMT and the role of ROS in the regulation of epidermal cells under hypoxia have not been investigated.

This study was aimed at determining the relationship between hypoxia and keratinocyte EMT and deciphering the underlying mechanisms. Additionally, we investigated whether ROS were involved in the regulation of EMT induced in hypoxic keratinocyte cells.

## 2. Materials and Methods

### 2.1. Mice and Ethics Statement

All animal studies followed the NIH Guide for the Care and Use of Laboratory Animals (NIH Pub. No. 85–23, revised 1996) and were approved by the research Ethics Committee of Shenzhen People's Hospital, China.

### 2.2. Cell and Mouse Skin Organ Culture

Immortalized human keratinocyte HaCaT cells were purchased from the Cell Bank of the Chinese Academy of Sciences (Shanghai, China), originally from American Type Culture Collection (Manassas, USA). The cells were cultured in a modified Eagle's medium (Gibco) containing 10% foetal bovine serum (FBS) and 1% penicillin and streptomycin (Gibco). Cells were maintained under standard cell culture conditions of 5% CO_2_ and 95% humidity at 37°C. Skin biopsies were derived from the dorsal skin of newborn C57BL/6 mice (postnatal days 1–3). Skin organ culture was performed as described in our previous report [5].

### 2.3. Hypoxia Exposure

Hypoxic conditions of 2% O_2_, 5% CO_2_, and 93% N_2_ were produced utilizing an oxygen control incubator (Smartor 118), which maintained a low O_2_ level under constant nitrogen flow at 37°C. The ROS scavenger NAC (A7250, Sigma, 5 mM), the *β*-catenin inhibitor XAV939 (Selleckchem, 5 *μ*M or 10 *μ*M), and acriflavine (ACF) (Sigma-Aldrich, 50 mM) were added to the cultures and incubated at 37°C during hypoxia treatment.

### 2.4. Western Blot Analyses

Whole-keratinocyte extracts and mouse skin specimens were first obtained for Western blot analyses and centrifuged at 14,000 rpm for 15 min at 4°C. The supernatants were then prepared, and protein concentrations were determined with a Bradford Protein Quantification Kit (500-0205, Bio-Rad Laboratories). The protein extracts were separated by SDS-PAGE and then transferred to polyvinylidene fluoride (PVDF) membranes (Millipore). The membranes were incubated overnight at 4°C with corresponding primary antibodies and secondary antibodies and visualized by the ChemiDoc XRS system (Bio-Rad Laboratories). Primary antibodies against the following were used to detect protein expression: HIF1 (20960-1-AP, 1 : 5000, Proteintech), vimentin (10366-1-AP, 1 : 5000, Proteintech), E-cadherin (20874-1-AP, 1 : 5000, Proteintech), N-cadherin (22018-1-AP, 1 : 5000, Proteintech), sex-determining region Y-box 2 (SOX2) (ab79351, 1 : 2000, Abcam), Slug (#9585, 1 : 1000, CST), *β*-catenin (#8480, 1 : 1000, CST), LEF1 (ab137872, 1 : 1000, Abcam), LGR5 (ab75850, 1 : 1000, Abcam), and *β*-actin (#3700, 1 : 1000, CST). Western blot analyses were performed as described in our previous report [12]. Quantified data were analysed using NIH ImageJ analytical software (http://rsb.info.nih.gov/ij/).

### 2.5. Cell Morphology

An inverted phase-contrast microscope was used to obtain representative images from HaCaT cells subjected to hypoxia treatment or normoxia treatment.

### 2.6. Immunofluorescence (IF) Staining

After treatment, the samples (cells cultured on glass coverslips and frozen mouse skin sections) were fixed in 4% paraformaldehyde for 20 min. The samples were incubated with primary antibodies at 4°C overnight and washed three times with phosphate-buffered saline (PBS). Next, the samples were stained with fluorescent secondary antibodies for 1 h at 37°C. Primary antibodies against the following were used: vimentin (10366-1-AP, 1 : 200, Proteintech), E-cadherin (20874-1-AP, 1 : 200, Proteintech), N-cadherin (22018-1-AP, 1 : 200, Proteintech), SOX2 (ab79351, 1 : 100, Abcam), and Slug (#9585, 1 : 1000, CST). The following secondary antibodies were used: FITC-labelled goat anti-rabbit IgG (F9887, 1 : 500, Sigma) and DyLight 594-labelled goat anti-mouse IgG (ab96881, 1 : 500, Abcam). The nuclei were then counterstained with DAPI (Abcam) before imaging. IF images were captured using fluorescence microscopy (Leica Microsystems, Germany).

### 2.7. Scratch Assay

HaCaT cells were plated on 6-well plates. Cell monolayers were scratched with 200 *μ*l plastic pipets. At 12 hours after wounding, cells that migrated into the cell-free area were monitored with an inverted light microscope (Olympus, Japan). Cell migratory ability was defined as the relative migrating area (%), which was measured using NIH ImageJ software.

### 2.8. ROS Detection

After various treatments, ROS levels were detected with a fluorescence microscope using the redox-sensitive fluorescent dye 2′,7′dichlorofluorescein (DCFH-DA; S0033S-1, Beyotime). Cells and frozen skin sections were incubated with DCFH-DA (10 *μ*M) for 20 min at 37°C. The samples were quickly washed twice with PBS to remove the extra dye and then detected and imaged under a fluorescence microscope. The fluorescence images were observed with a microplate reader, and fluorescence was measured with a fluorescence microplate reader (SPARK 10 M, Tecan) with an excitation wavelength of 488 nm and emission wavelength of 525 nm for DCFH-DA.

### 2.9. Quantitative Real-Time Reverse Transcription PCR (qRT-PCR)

Total RNA was extracted by AG RNAex Pro Reagent (AG21102, Accurate Biotechnology, Hunan). The amount of RNA extracted was measured with OD260/OD280 ratios between 1.9 and 2.1, and the total isolated RNA was reverse transcribed into cDNA using the PrimeScript RT reagent Kit (RR037A, Takara, Japan). Real-time PCR was performed using TB Green Premix Ex Taq (RR420A, Takara, Japan) on a StepOnePlus quantitative PCR system (Applied Biosystems, USA). The primer sequences used for qRT-PCR are listed in Supplementary Table S1. As an internal control, the levels of *β*-actin were quantified in parallel with the levels of target genes. Normalization and fold changes were calculated by the comparative *ΔΔ*CT method.

### 2.10. siRNA Cell Transfection

On the day prior to transfection, cells were plated to the required cell density (at least 70% confluency). Small interfering RNAs (siRNAs) specific for SOX2 and the corresponding scrambled siRNA (as a control; siCtrl) were individually diluted in Opti-MEM (Life Technologies) and incubated for 5 min at room temperature. The diluted siRNAs were added to diluted Lipofectamine 2000 (11668, Invitrogen) and further incubated for 20 min. The complex was added according to the manufacturer's protocol. The above sequences used for siRNA cell transfection are listed in Fig. S1.

### 2.11. Luciferase Assays

HEK293T cells were transfected with a DNA mixture containing the SOX2 expression plasmid pLV-SOX2, pGL3-enhancer containing the *β*-catenin promoter, and pRL-TK plasmid. After 30 h, the Dual-Luciferase Reporter Assay System (Promega) was used to test relative luciferase and Renilla activities according to the manufacturer's instructions.

### 2.12. Chromatin Immunoprecipitation-PCR (ChIP-PCR)

Hypoxia-treated cells (4 × 10^6^) were cross-linked by 1% formaldehyde treatment. Glycine was used to stop the cross-linking. Chromatin was sonicated and sheared into small fragments following the instructions of the EZ-ChIP™ kit (17-371, Millipore, Billerica, MA). One microgram of anti-RNA polymerase (05-623, Millipore, Billerica, MA), 1 *μ*g of IgG (ab96881, Abcam), or 1 *μ*g of anti-SOX2 antibody (ab79351, Abcam) was added to pull down the target protein. Target protein-bound DNA was purified and subjected to PCR amplification of an ~200 bp fragment of the *β*-catenin promoter using the following primer sequences: FW: 5′-GCCGAGTGGAAACTTTTGTCG-3′, BW: 5′-GGCAGCGTGTACTTATCCTTCT-3′.

### 2.13. *In Vivo* Wound Closure Assay

Male and female C57BL/6 mice (8 to 12 weeks old) were anaesthetized through intraperitoneal administration of sodium pentobarbital, and their hair was shaved with 70% ethanol. Full-thickness wounds were created on the middorsal skin with 8 mm disposable biopsy punches as described in our previous report [13]. To confirm the effect of ROS accumulation during cutaneous wound healing in mice, 100 mg/kg NAC dissolved in 200 ml of saline or the same volume of saline (as a control) was topically applied to the dorsal mouse skin daily until the wound resolved. For further analysis, each wound site was digitally photographed, and wound tissue was harvested at the indicated time points after wounding. Photographs of the wound areas were assessed by NIH ImageJ software. Alterations in the wound area are reported as the percentage of the initial wound area in mice.

### 2.14. Histological and Immunohistochemistry Staining

Skin tissues were excised, fixed in 4% paraformaldehyde, embedded and sectioned in paraffin, and stained with haematoxylin and eosin (H&E) (Sigma, Poole, UK). Digital images were taken by light microscopy (Olympus BX51, Olympus, Japan).

Immunohistochemical analysis was used to determine the expression of Snail and Slug. Formalin-fixed paraffin-wax-embedded skin biopsies were sectioned (4 mm) and incubated with antibodies against vimentin (10366-1-AP, 1 : 2000, Proteintech) and N-cadherin (22018-1-AP, 1 : 2000, Proteintech). The antibodies were detected using 3,3′-diaminobenzidine tetrahydrochloride to create a brown reaction product, counterstained with haematoxylin (Dako, Ely, UK), and examined by light microscopy.

### 2.15. Statistical Analyses

All experiments were performed at least in triplicate to be eligible for the indicated statistical analysis. Statistical comparison was then performed with GraphPad Prism 5.0 (GraphPad Software Inc.). All data were presented as the mean ± SEM. The Shapiro-Wilk test was used to check whether the data were normally distributed. Differences in the results between two groups were analysed using a two-tailed unpaired *t*-test if the data satisfied the normality requirement. A two-tailed Wilcoxon-test was used to test variances between the two groups if the data did not satisfy the normality requirement. Statistical significance among three or more groups was assessed by one-way analysis of variance (ANOVA). Differences between two or more groups at different time points were estimated using two-way ANOVA. The Bonferroni method was used for multiple-group comparisons post-ANOVA. A *P* value of <0.05 was used to indicate statistical significance (^∗^*P* < 0.05, ^∗∗^*P* < 0.01, ^∗∗∗^*P* < 0.001, and ^∗∗∗∗^*P* < 0.0001).

## 3. Results

### 3.1. Hypoxia Induced EMT and an Increase in the Motility of HaCaT Cells

**Figure 1:**
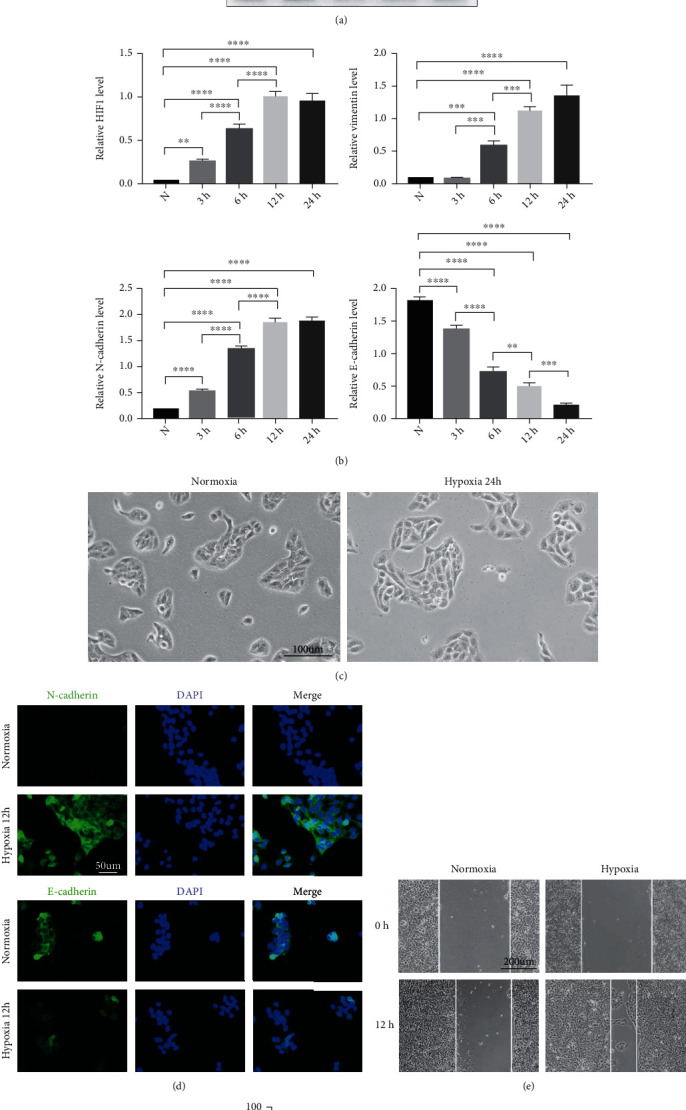
Hypoxia induced EMT and increased the motility of HaCaT cells. (a, b) Western blotting (a) and quantitative analysis (b) were employed to analyse the expression levels of HIF1, vimentin, N-cadherin, and E-cadherin in normoxia-treated (N) and hypoxia-treated HaCaT cells at the indicated time points. *β*-Actin was used as the loading control. (c) Representative pictures of change in cell phenotype in normoxia-treated and hypoxia-treated HaCaT cells up to 24 h are shown. Bar, 100 *μ*m. (d) Representative images of fluorescence staining of the indicated HaCaT cells. Bar, 50 *μ*m. (e, f) Scratch assays (e) and quantitative analysis (f) were performed using the indicated cells under hypoxia or normoxia for 12 h. Bar, 200 *μ*m. Mean ± SEM. *n* = 3. ^∗∗^*P* < 0.01, ^∗∗∗^*P* < 0.001, and ^∗∗∗∗^*P* < 0.0001.

To determine the effect of hypoxia on human keratinocytes, HaCaT cells were selected and treated with hypoxia as described in our previous study [5, 10]. Next, we tested HIF1 expression in HaCaT cells to confirm the establishment of a hypoxic model (2% O_2_) (Figures 1(a) and 1(b)). Then, changes in HaCaT cell shape in response to hypoxia treatment were assessed. After 24 h of hypoxia exposure, the cells presented changes such as an elongated phenotype and decreased density (Figure 1(c)). Because the acquisition of a spindle-like shape in epithelial cells is recognized as a hallmark of EMT, we further studied the expression levels of EMT markers to verify cell changes consistent with EMT. EMT is characterized by the repression of E-cadherin expression and induction of N-cadherin and vimentin expression [14]. As shown in Figures 1(a) and 1(b), HaCaT cells showed significantly increased expression of vimentin and N-cadherin and decreased expression of E-cadherin in a time-dependent manner under hypoxic conditions compared with normoxic conditions (21% O_2_). IF analysis revealed that the expression levels of N-cadherin increased and those of E-cadherin decreased, as accompanied by a long and fibroblast-like shape, after 12 h of treatment with hypoxia (Figure 1(d)), when the hypoxia-induced changes in E-cadherin, N-cadherin, and vimentin expression compared to their expression under normoxia were most obvious (Figures 1(a) and 1(b)). In addition, a scratch assay revealed that hypoxia increased more cell migration compared with that under normoxia (Figures 1(e) and 1(f)). These above results confirm the effect of hypoxia in promoting EMT and motility in HaCaT cells.

### 3.2. Hypoxia Increased EMT and Migration by ROS

**Figure 2:**
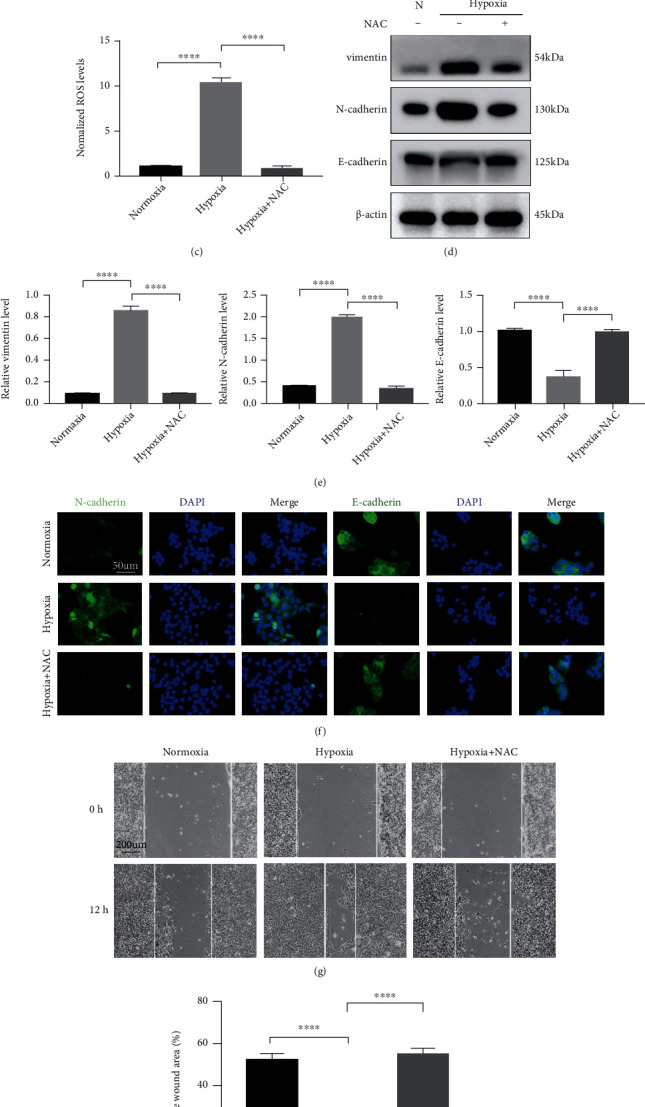
ROS are required for hypoxia-stimulated EMT and migration of HaCaT cells. (a–c) Levels of endogenous ROS in HaCaT cells were measured by staining with DCFH-DA. Fluorescent DCFH-DA was monitored at the indicated time points by a microplate reader (a, c). Representative pictures showing staining with DCFH-DA (b). (d, e) Western blotting (d) and quantitative analysis (e) were performed to detect vimentin, N-cadherin, and E-cadherin levels with or without hypoxia and NAC treatment for 12 h. *β*-Actin was used as the loading control. (f) Representative fluorescent images of N-cadherin and E-cadherin in the indicated HaCaT cells. Bar, 50 *μ*m. (g, h) Scratch assays (g) and quantitative analysis (h) were performed using NAC-treated and untreated HaCaT cells with or without hypoxia exposure for 12 h. Bar, 200 *μ*m. Mean ± SEM. *n* = 3. ^∗∗∗^*P* < 0.001; ^∗∗∗∗^*P* < 0.0001.

Hypoxia is associated with the generation of ROS [15], consistent with our results (Figures 2(a) and 2(b)). An MTS assay was employed to evaluate the effect of ROS on keratinocyte viability. To that end, HaCaT cells were cultured for 24 h under hypoxia with or without NAC treatment. After 24 h of exposure, none of the treated cells showed a change in cell viability, regardless of whether ROS were present (Fig. S2A-B). We further demonstrated the role of ROS in other process, including the EMT process, upon the acquisition of increased migratory potential in HaCaT cells by inhibitor treatment. Normalized ROS levels with or without hypoxia and NAC treatment for 12 h were assessed with a fluorescence microplate reader (Figure 2(c)). As shown in Figures 2(d)–2(f), the reduction of ROS induced by NAC greatly attenuated hypoxia-induced EMT. Likewise, we found that the complete absence of intracellular ROS accumulation induced by NAC abrogated the hypoxia-stimulated increase in the migratory capacity of HaCaT cells (Figures 2(g) and 2(h)). These results indicate the positive role of ROS in hypoxia-triggered EMT and the increased migratory capacity in HaCaT cells.

### 3.3. SOX2 Is Required for ROS-Driven EMT and Migration under Hypoxic Conditions

**Figure 3:**
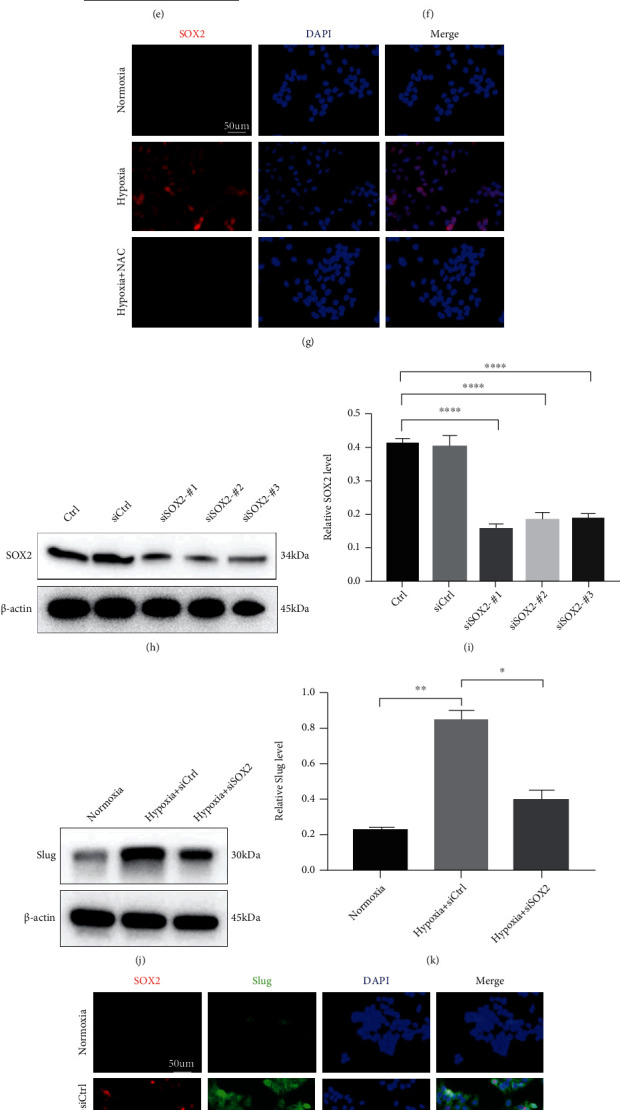
The effect of SOX2 on ROS-induced HaCaT cell EMT and migration under hypoxic conditions. (a, b) HaCaT cells were incubated under hypoxia for the indicated times. SOX2 expression was assayed using Western blotting (a). Quantitative analysis (b) was employed to analyse the relative SOX2 level. SOX2 was quantified and normalized against *β*-actin. (c) Representative pictures of IF staining for SOX2 expression in cells treated with hypoxia or normoxia for 12 h. Bar, 50 *μ*m. (d–f) The mRNA (d) and protein (e, f) levels of SOX2 were analysed in NAC-treated and untreated HaCaT cells with or without hypoxia treatment. (g) Representative fluorescent images of SOX2 in the indicated HaCaT cells. Bar, 50 *μ*m. (h, i) The protein extracts from hypoxia-treated cells transfected with siSOX2 or siCtrl were analysed via Western blotting (h) and quantitative analysis (i) for SOX2. (j, k) Western blotting (j) and quantitative analysis (k) were performed to detect Slug levels in cells transfected with siSOX2 or siCtrl after 12 h of treatment with hypoxia or normoxia. (l) The expression of SOX2 (red) and Slug (green) was determined by IF staining. The representative images show typical coexpression of SOX2 and slug. Bar, 50 *μ*m. (m, n) Scratch assays (m) and quantitative analysis (n) were performed using the indicated cells with or without hypoxia treatment for 12 h. Bar, 200 *μ*m. Mean ± SEM. *n* = 3. (o) Levels of endogenous ROS were measured by a microplate reader. Mean ± SEM. *n* = 3. ^∗^*P* < 0.05, ^∗∗^*P* < 0.01, ^∗∗∗^*P* < 0.001, and ^∗∗∗∗^*P* < 0.0001.

The transcription factor SOX2 is generally considered a stemness marker. SOX2 has also been shown to function downstream of ROS signals [16–18], and SOX2 is involved in hypoxia-associated modulation of cancer cell invasion, migration, and EMT [19, 20]. Therefore, we then detected the expression of SOX2 in hypoxia-exposed HaCaT cells over time. By Western blot analyses, we found SOX2 expression to be greatly elevated in a time-dependent manner under hypoxic conditions for 3, 6, 12, and 24 h compared with that under normoxic conditions (Figures 3(a) and 3(b)). IF analysis of representative pictures revealed similar changes with hypoxia treatment for 12 h, as shown in Figure 3(c). In addition, further experiments were designed to examine the association between SOX2 and ROS in hypoxia-stimulated HaCaT cells. Interestingly, the mRNA and protein levels of SOX2 after hypoxia exposure was significantly inhibited by treatment with NAC (Figures 3(d)–3(f)). Representative fluorescent images showing SOX2 expression are provided in Figure 3(g). Collectively, the above results revealed that SOX2 acted downstream of ROS in HaCaT cells subjected to hypoxia.

To determine the role of SOX2 in EMT and the promigration effect of ROS under hypoxia, SOX2 expression was knocked down with appropriate siRNAs and examined by Western blot analysis. Compared with siCtrl, siRNA 3 against SOX2 (3 siSOX2) remarkably downregulated SOX2 protein levels in HaCaT cells (Figures 3(h) and 3(i)), and the most effective siSOX2-#1 was selected for the follow-up study. We further detected the expression of Snail and Slug in cells. As zinc-finger transcription factors, Snail and Slug are the dominant players that orchestrate orchestrating EMT by repressing E-cadherin and increasing N-cadherin [21]. As illustrated in Figures 3(j) and 3(k), under hypoxia treatment for 12 h, SOX2-silenced HaCaT cells showed a reduction in Slug expression compared to that in HaCaT cells transfected with siCtrl, whereas no expression of Snail was detected (unshown). Moreover, coexpression of SOX2 and Slug was ubiquitous in HaCaT cells subjected to hypoxia compared with cells subjected to normoxia, as shown in Figure 3(l). Knockdown of SOX2 suppressed the hypoxia-induced increase in the migration of HaCaT cells subjected to hypoxia (Figures 3(m) and 3(n)). The above results demonstrated that silencing SOX2 inhibited hypoxia-stimulated EMT and increased HaCaT cell migration. Meanwhile, SOX2-deficient HaCaT cells responded to hypoxia with no change in ROS expression compared with that in cells transfected with siCtrl (Figure 3(o)). Overall, the above data revealed a previously unknown mechanism by which hypoxia upregulates ROS/SOX2 signalling.

### 3.4. Wnt/*β*-Catenin Is a Downstream Pathway of SOX2 under Hypoxia

**Figure 4:**
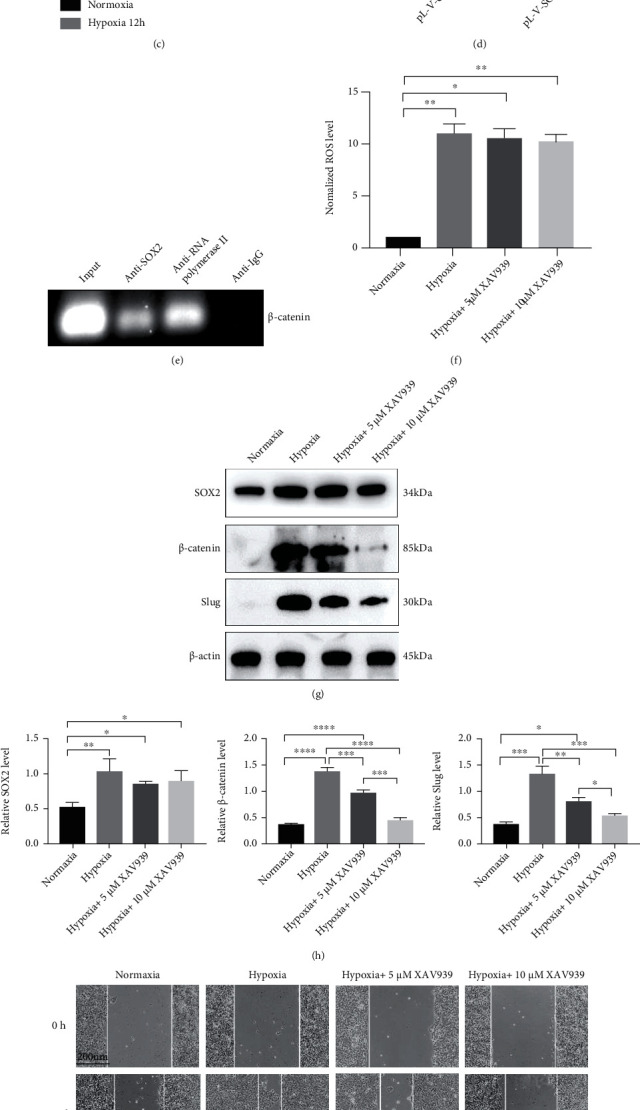
Validation of the Wnt/*β*-catenin pathway as a target of SOX2 in HaCaT cells under hypoxia. (a) qRT-PCR was employed to detect the mRNA levels of PORCN, WNT11, FZD5, BAMBI, FOSL1, CTNNB1 (*β*-catenin gene), LEF1, and LGR5 in HaCaT cells subjected to hypoxia for 12 h. (b, c) Western blotting (b) and quantitative analysis (c) were performed to detect *β*-catenin, LEF1, and LGR5 levels in cells after 12 h of treatment with hypoxia or normoxia. *β*-Actin was used as the loading control. (d) Luciferase assays were used to analyse activation of the *β*-catenin promoter upon SOX2 overexpression in HaCaT cells. (e) ChIP-PCR was employed to uncover the binding of SOX2 to the *β*-catenin promoter in HaCaT cells. (f) Levels of endogenous ROS were measured by a microplate reader. (g, h) Western blotting (g) and quantitative analysis (h) of *β*-catenin, Slug, and SOX2 were carried out in HaCaT cells with or without hypoxia and XAV939 treatment for 12 h. (i, j) Scratch assays (i) and quantitative analysis (j) were performed using XAV939-treated and untreated HaCaT cells with or without hypoxia treatment for 12 h. Bar, 200 *μ*m. Mean ± SEM. *n* = 3. ^∗^*P* < 0.05, ^∗∗^*P* < 0.01, ^∗∗∗^*P* < 0.001, and ^∗∗∗∗^*P* < 0.0001.

Although a previous study reported that Slug is a direct transcriptional target of SOX2 [22], we failed to detect strong binding of SOX2 to the Slug promoter after hypoxia treatment by luciferase reporter assay (unshown). Thus, it is likely that SOX2-stimulated Slug induction is indirect. To test this hypothesis, we identified SOX2 target genes that mediate its stimulatory activity. Uchiyama et al. generated epidermal-specific SOX2-overexpressing mice and analysed changes in the gene expression in skin wounds with respect to their expression in control mice [23]. Kyoto Encyclopedia of Genes and Genomes (KEGG) enrichment analysis of the upregulated mRNAs revealed the top ten enriched canonical pathways (Fig. S3A). Among these pathways, the Wnt signalling pathway is known to mediate EMT [24]; thus, the Wnt pathway is likely to be downstream of SOX2 in the regulation of hypoxia-induced EMT. To test this assumption, the mRNA levels of PORCN, WNT11, FZD5, BAMBI, FOSL1, CTNNB1 (*β*-catenin gene), LEF1, and LGR5, which were enriched in the Wnt pathway according to KEGG pathway analysis, were first detected by qRT-PCR in hypoxia-mediated HaCaT cells (Fig. S3B). As illustrated in Figures 4(a)–4(c), the mRNA and protein expression of *β*-catenin, LEF1, and LGR5 was remarkably increased in HaCaT cells after 12 h of hypoxia exposure compared with normoxia. The dual-luciferase assay further revealed increased activation of the *β*-catenin promoter by SOX2 when compared with that in controls (Figure 4(d)). In addition, ChIP-PCR revealed that SOX2 can directly bind the *β*-catenin promoter and upregulate *β*-catenin expression (Figure 4(e)). Next, HaCaT cells were treated with XAV939, which selectively promotes *β*-catenin degradation by stabilizing Axin1 and suppresses the Wnt/*β*-catenin pathway [25]. As shown in Figures 4(f)–4(h), the expression levels of both *β*-catenin and Slug were inhibited in HaCaT cells subjected to hypoxia, unlike the SOX2 expression level and ROS generation. Likewise, the migratory potential of HaCaT cells was decreased in a dose-dependent manner (Figures 4(i) and 4(j)). These results demonstrate that activation of the Wnt/*β*-catenin pathway is required for ROS/SOX2-induced EMT and migration in hypoxia-exposed HaCaT cells.

### 3.5. Hypoxia-Induced ROS Production Is Required for Epidermal SOX2 and *β*-Catenin Expression and EMT *In Vivo*

**Figure 5:**
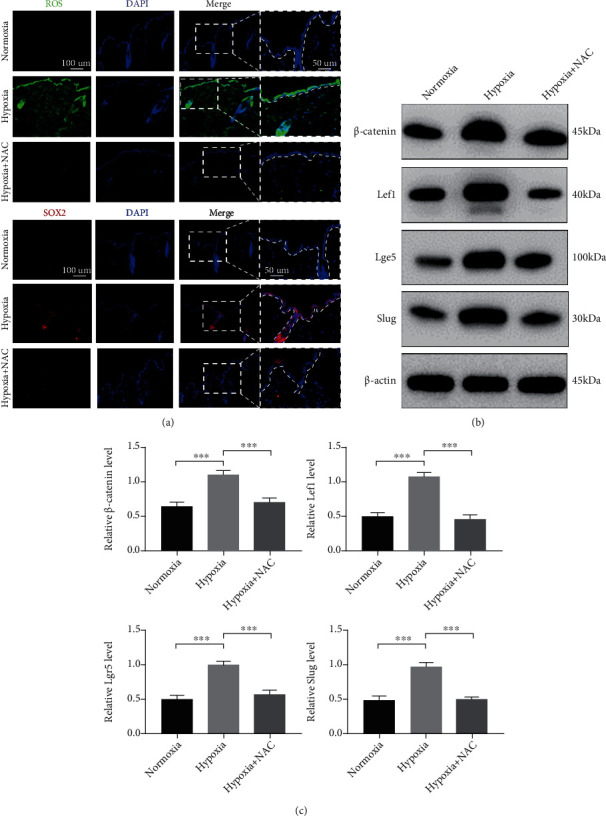
Increased ROS production induced by hypoxia drove epidermal SOX2 and *β*-catenin expression and EMT *in vivo*. (a) The levels of ROS (green) and expression of SOX2 (red) were determined by IF staining. Representative images show typical ROS and SOX2 levels. The magnification of the dotted boxes is shown on the right. Additionally, the epithelium is marked with a dotted line. Bar, 100 and 50 *μ*m. Western blotting (b) and quantitative analysis (c) were performed to analyse the expression of *β*-catenin, Lef1, Lgr5, and Slug in cultured skin subjected to hypoxia with NAC. *β*-Actin was used as the loading control. Mean ± SEM. *n* = 3. ^∗^*P* < 0.05, ^∗∗^*P* < 0.01, ^∗∗∗^*P* < 0.001, and ^∗∗∗∗^*P* < 0.0001.

Wound-induced accumulation of ROS is triggered by a hypoxic microenvironment [26]. To determine whether hypoxia-derived ROS mediate SOX2 expression and EMT *in vivo*, we utilized an *in vitro* culture model of the skin from the dorsal coat of newborn C57BL/6 mice to mimic the hypoxic microenvironment as described previously [5]. The skin specimens were cultured in 6-well plates under hypoxia for 6 h with or without NAC treatment. IF and Western blot analyses showed that hypoxia-induced ROS production triggered SOX2 expression in the epidermis (Figure 5(a)), which was attenuated by the addition of NAC. Similarly, the expression of *β*-catenin, Lef1, Lgr5, and Slug was remarkably upregulated in the epidermis subjected to hypoxia (Figures 5(b) and 5(c)), and the expression of all was inhibited by the addition of NAC. Collectively, these results indicate that hypoxia-derived ROS production is required for SOX2 expression and Wnt/*β*-catenin pathway activation, which further induces Slug expression in tissue.

### 3.6. ROS Appear to Be Involved in EMT-Driven Reepithelialization during Repair

**Figure 6:**
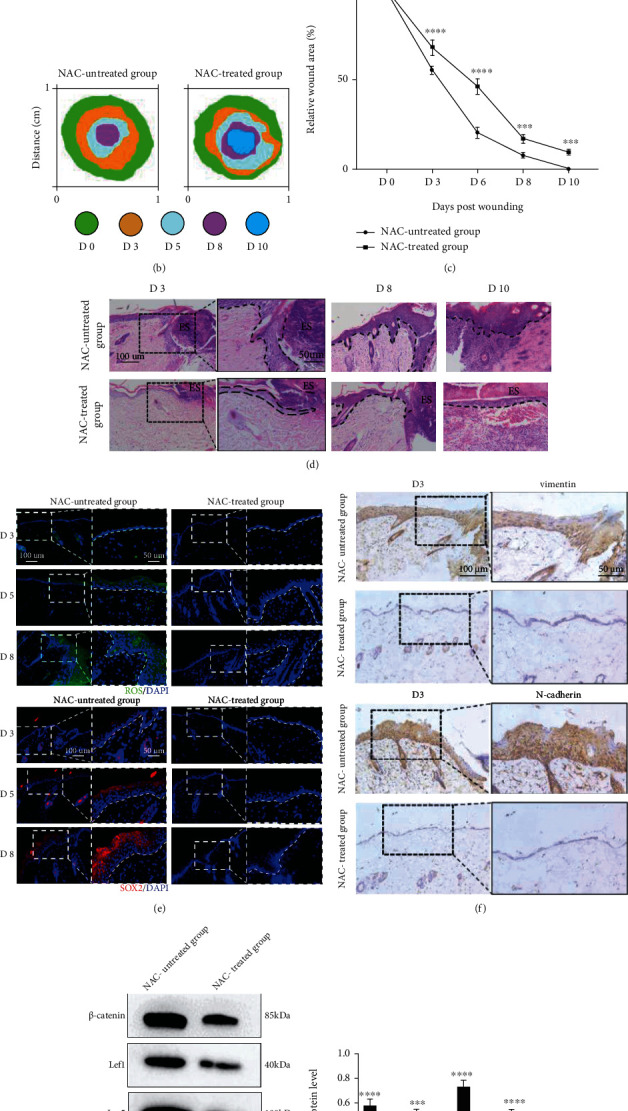
ROS are required for EMT-driven reepithelialization during repair. (a–c) Representative pictures and quantitation of the healing time of skin wounds after an 8 mm primary biopsy. Biopsy sites were demarcated using blue polypropylene sutures. Bar, 2 mm. Mean ± SEM. *n* = 6. (d) Representative H&E-stained pictures of skin wounds on days 3, 8, and 10. The magnification of the dotted boxes is shown on the right at day 3 after wounding. Additionally, the epithelium is marked with a dotted line. Bars, 100 and 50 *μ*m (ES: Eschar). (e) Representative pictures of wounded (days 3, 5, and 8) skin stained to show the expression of ROS (green) and SOX2 (red) in the skin (blue). The magnification of the dotted box is shown on the right of each picture. Additionally, the epithelium is marked with a dotted line. Bar, 100 and 50 *μ*m. (f) Immunohistochemical analysis of vimentin and N-cadherin in skin wounds on day 3. The magnification of the dotted boxes is shown on the right. Bars, 100 and 50 *μ*m. (g, h) Western blotting (g) and quantitative analysis (h) were employed to analyse the expression levels of *β*-catenin, Lef1, Lgr5, and Slug in the NAC-treated wounds and untreated wounds at day 3 after wounding. *β*-Actin was used as the loading control. Mean ± SEM. *n* = 3. ^∗∗∗^*P* < 0.001; ^∗∗∗∗^*P* < 0.0001.

To further investigate the effect of ROS in EMT-driven reepithelialization during repair, we generated a wound model of conditional inhibitory cutaneous production of ROS by NAC and analysed the wounded skin with and without ROS accumulation. Analysis of the healing time revealed that untreated mice exhibited significantly accelerated wound closure compared with that in the treated group from day 3 to day 10 (Figures 6(a)–6(c)). Histological analysis of the wound sections showed earlier reepithelialization of the untreated wounds compared to the treated wounds at day 3 after wounding, as well as significantly increased thickness of the epidermis in untreated mice compared to control mice at days 8 and 10 after wounding (Figure 6(d)). IF analysis further showed that wounding-activated ROS accumulated in the untreated wounds, whereas ROS were not detected in the NAC-treated wounds (Figure 6(e)). These results indicate that ROS generation in skin keratinocytes accelerates cutaneous wound healing by promoting reepithelialization.

Meanwhile, ROS accumulation significantly upregulated the expression of SOX2 during wound healing in the untreated wounds compared with the NAC-treated wounds (Figure 6(e)). EMT markers (vimentin and N-cadherin) were present in the epithelium of the wounded skin, and their levels were significantly increased in untreated wounds compared to NAC-treated wounds during wounding (Figure 6(f)). Wound-induced changes in the expression of *β*-catenin, Lef1, Lgr5, and Slug were obvious in the untreated wounds compared to the NAC-treated wounds at day 3 after wounding (Figures 6(g) and 6(h)). In summary, these results indicate that ROS aid in accelerating wound closure by reinforcing EMT-driven reepithelialization, which is mediated by the SOX2 and Wnt/*β*-catenin pathways.

## 4. Discussion

Our previous study demonstrated that wound-induced hypoxia is related to keratinocyte migration [5]. During wound healing, keratinocytes are generally thought to undergo EMT for the acquisition of migratory properties. Additionally, hypoxia has recently been identified as a critical player in the cellular expression program that thus enhances cancer cell EMT [6]. However, the role of wound-induced hypoxia in driving the EMT of nontumorigenic keratinocytes and potential mechanism are poorly documented. In the present study, we found that the EMT phenotype was concomitantly acquired in migrating epidermal cells in a hypoxia-dependent manner. Additionally, our work has revealed a mechanism through which hypoxia promotes ROS, which participates in inducing keratinocyte EMT.

Insufficient blood supply to wound tissue and heightened oxygen consumption lead to diminished oxygen availability. In fact, the O_2_ concentration in a hypoxic environment, where a large proportion of cells around the damaged area exist, is 2% O_2_, and this level was adopted in our previous studies [5, 10]. In this study, we also used 2% O_2_ to establish a hypoxic model. It is well known that hypoxia positively alters cellular metabolism to help cells adapt to a low-oxygen environment. Among these alterations, an increased ROS-producing capacity was detected in response to low-oxygen content via the activation of reductive carboxylation [7, 27, 28]. In agreement with these studies, our results also showed that ROS production was upregulated by hypoxia treatment. Nevertheless, ROS seem to act as a double-edged sword in terms of cell fate. ROS are regarded as highly reactive molecules that are responsible for damaging cellular components [29]. ROS overproduction leads to cell death by provoking the destruction of intracellular DNA, lipids, and macromolecules [30]. In this study, we evaluated the role of ROS in keratinocyte viability by MTS assay, the results of which indicated that the keratinocytes responded to hypoxia-induced ROS with no change in cell viability compared with viability in the complete absence of intracellular ROS through NAC treatment, suggesting that hypoxia-induced ROS were not toxic to the keratinocytes. It is postulated that variation in ROS formation may be responsible for the different effects of ROS on cell apoptosis. The current data confirmed that a low-oxygen (2% O_2_) environment simply maintains ROS production at a low physiological level, avoiding uncontrolled ROS formation. Here, our results provide a concept for the existence of a mechanism underlying the maintenance of hypoxia-induced ROS at a balanced level in keratinocytes subjected to moderate hypoxic conditions (2% O_2_), which requires further investigation.

In addition to the role of ROS harmful factors, ongoing studies have revealed that ROS appear to be important signal mediators involved in physiological processes in the cell [31, 32]. Considering that ROS production is linked to a low-oxygen environment, we hypothesized that hypoxia-induced ROS production is the earliest biochemical limiting factor of keratinocyte activation in a hypoxia-dependent manner. Our previous studies indicated that a reduction in hypoxia-induced ROS through NAC attenuated the galvanotaxis of keratinocytes [10], but the underlying mechanisms have remained largely unclear. The present data seem to preliminarily confirm that hypoxia-induced ROS generation is an essential element for regulating keratinocyte EMT. EMT is characterized by the disappearance of cell polarity and decreased cell adhesion, and this process primes original stationary keratinocytes for the acquisition of migratory potential [3], which enhances the galvanotaxis of keratinocytes.

Apart from the role of ROS in ensuring hypoxia-mediated EMT and migration, we further explored the mechanism by which ROS promote keratinocyte EMT and migration under hypoxia. According to the literature, low levels of intracellular ROS, as second messengers, induce various downstream signalling effects that contribute to the regulation of cell fate [33]. Recent work has suggested that SOX2 functions downstream of ROS signals [18]. In addition, recent reports have revealed that hypoxia increases the expression of SOX2 [34, 35]. Meanwhile, SOX2 has been shown to regulate networks responsible for accelerated wound resolution [36]. However, to date, the relationship between hypoxia-triggered ROS and SOX2 and the mechanistic role of SOX2 in the regulation of wound repair have remained unknown. Thus, it is necessary to explore the link between ROS and SOX2 and the role of SOX2 in hypoxia-stimulated EMT and keratinocyte migration. In line with previous studies, our study showed that hypoxia-induced upregulation of SOX2 expression was attenuated after ROS scavenging. Notably, several studies reported the ability of HIF1 to induce SOX2 in cancer cells [37], and HIF1 acted as an oxygen sensor in the present study. To determine whether HIF1 also plays a role in the regulation of SOX2 expression, we assessed SOX2 expression in keratinocytes treated with ACF due to its direct inhibition of HIF-1 activation [38] and its cellular safety [39]. Interestingly, we found that the inhibition of HIF1 expression did not affect the increased expression of SOX2 in keratinocytes in a hypoxia-dependent manner (Fig. S4A-B). On the basis of our study, hypoxia-induced SOX2 activation is suggested to be associated with ROS production in keratinocytes. ROS are important messengers that promote the expression of stemness-associated SOX2 in response to oxidative stress [40]. However, in contrast, other reports have mentioned the attenuated inductive effect of SOX2 under a high ROS concentration [41, 42]. This suggests that the disruption of stemness-associated SOX2 expression may be closely related with excessive ROS formation, since ROS above a certain concentration threshold could inhibit the self-renewal of stem cells [43]. Collectively, the results also imply the role of physiologically low ROS levels in driving SOX2 expression in hypoxia-treated keratinocytes.

Although it is also well known that SOX2 plays a critical role in stabilizing the pluripotency of adult stem cells [44], there is growing evidence that SOX2 positively affects cell features such as the capacity to invade and migrate and EMT [45, 46]. Our current findings also indicated that ROS-induced activation of EMT and migration was attenuated by SOX2 silencing in cells subjected to hypoxia. Furthermore, the activity of SOX2 contributes to context-dependent expression of Slug [47, 48], which is known to drive EMT by downregulating E-cadherin and upregulating N-cadherin [49]. We further provide evidence that SOX2 inhibition in keratinocytes exposed to hypoxia reduced the expression levels of Slug. Notably, in addition to ensuring cell motility, EMT is involved in restoring self-renewal properties [3], indicating that the role of SOX2 in stimulatory keratinocyte EMT might be linked to its stem cell regulatory functions. Future investigations are needed to determine whether SOX2 derived from hypoxic keratinocytes also functions to maintain stemness in cells.

The transcription factor SOX2 contributes to the regulation of gene transcription, thereby mediating cell fate as well as axis and pattern formation [50]. Slug has been reported to be a direct transcriptional target of SOX2 [22]. However, we did not detect strong binding of SOX2 to the Slug promoter after hypoxia treatment, suggesting that SOX2 indirectly mediates Slug induction in keratinocytes. Epidermal-specific SOX2 overexpression was reported to result in increased expression of genes associated with keratinocyte differentiation, EMT, and keratinocyte movement by gene expression analysis [23]. KEGG analysis further showed that genes whose expression was significantly increased were enriched in the Wnt pathway, which is known to play an exceedingly important role in regulating EMT [24]. Thus, we speculated that activation of the Wnt pathway is associated with SOX2-mediated EMT in hypoxic keratinocytes. Increased expression of Lef1, Lgr5, and *β*-catenin was observed in keratinocytes under hypoxia. LEF1 is a *β*-catenin-responsive gene, and the stem marker Lgr5 is a target gene of *β*-catenin transcriptional activity [51]. Additionally, previous studies have demonstrated the regulatory effect of SOX2 on *β*-catenin expression [52]. Hence, we further explored the link between SOX2 and *β*-catenin in hypoxia-exposed keratinocytes. Through luciferase-based gene reporter and ChIP-PCR assays, *β*-catenin was demonstrated to be a potential molecular target of SOX2 in keratinocytes under hypoxic conditions. Utilizing selective inhibition, we further showed that keratinocyte EMT and migration under hypoxia are mediated by SOX2-induced Wnt/*β*-catenin activation. Based on the above results, we assumed that SOX2 exerted a stimulatory effect by activating the Wnt/*β*-catenin pathway in hypoxic keratinocytes. Meanwhile, the present study helps advance our knowledge of the role of the Wnt pathway in keratinocyte EMT, which is rarely reported and requires further investigation.

Finally, we explored the potential mechanism by which wound-induced ROS regulate skin repair. First, an *ex vivo* skin culture model in response to hypoxia was employed. Further results demonstrated the important role of ROS in the enhancement of SOX2 expression and Wnt/*β*-catenin pathway activation, thereby enhancing Slug expression in tissue. Topical application of low doses of ROS was demonstrated to enhance wound closure in mice [53]. Here, we also found that the removal of ROS resulted in delayed wound closure. Previous experiments have revealed EMT as a crucial facilitator of wound-triggered migratory capacity in epithelial cells [3]. In addition, the movement of epithelial cells is thought to play a critical role in ensuring reepithelialization and proper wound healing. Consequently, we determined the levels of EMT markers and reepithelialization in wound repair. The results demonstrated that wound-induced ROS promoted the induction of EMT and subsequent rapid reepithelialization of the wound area, but these effects were inhibited by NAC. In addition, further results suggested that SOX2 expression and subsequent activation of Wnt/*β*-catenin are involved in ROS-associated modulation of reepithelialization of the wound area. In a further study, we will investigate whether the regulation of SOX2 can affect the activation of Wnt/*β*-catenin and thereby reepithelialization in wound healing.

## 5. Conclusion

**Figure 7:**
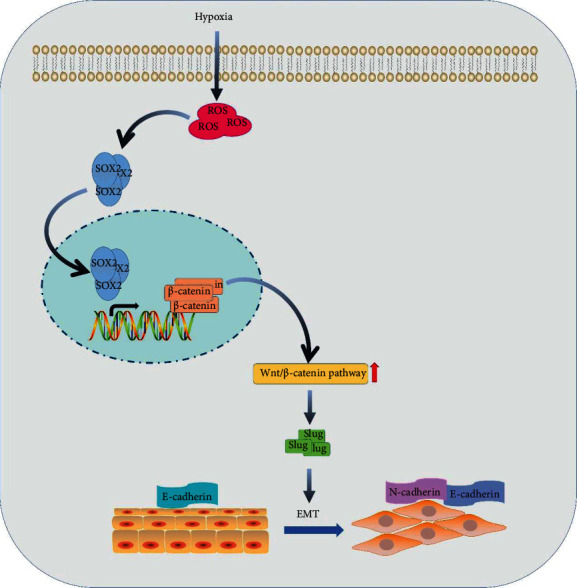
Schematic of keratinocyte activation by hypoxia treatment: the primary event in this process is the production of ROS in response to hypoxia treatment. ROS function as secondary messengers and stimulate SOX2 expression and subsequent activation of Wnt/*β*-catenin in HaCaT cells. The activation of Wnt/*β*-catenin, in turn, induces EMT, accelerates migration, and promotes wound healing.

In conclusion, our study documents for the first time the novel role of ROS in the determination of EMT and movement via the enhancement of SOX2 and activation of Wnt/*β*-catenin in hypoxic keratinocytes (Figure 7). We further present evidence that hypoxia promotes wound healing by promoting reepithelialization through ROS production accompanied by SOX2 expression and Wnt/*β*-catenin pathway activation. These findings support strategies to target these relevant molecules and pathways for improving wound healing.

## Data Availability

The raw experimental data used to support the findings of this study are available from the corresponding author upon request.

## Supplementary Materials

Supplementary Materials Figure S1: the sequences of primers and siRNAs used for qRT-PCR and siRNA cell transfections, respectively. Figure S2: hypoxia-triggered ROS did not affect cell viability. HaCaT cells were treated with hypoxia and NAC over a series of time points before analysis. (A) Levels of endogenous ROS were measured by a microplate reader. (B) Cell viability was determined by MTS assays. Mean ± SEM. *n* = 3. ^∗∗∗^*P* < 0.0001. Figure S3: KEGG enrichment analysis of upregulated mRNAs (A) and the enrichment of upregulated mRNAs in the Wnt pathway (B). Figure S4: Western blotting (A) and quantitative analysis (B) were employed to analyse the expression levels of HIF1 and SOX2 in hypoxia-treated HaCaT cells with or without ACF treatment for 12 h. Mean ± SEM. *n* = 3. ^∗∗∗^*P* < 0.0001.
