# Supplementary material for: ROS Promote Hypoxia-Induced Keratinocyte Epithelial-Mesenchymal Transition by Inducing SOX2 Expression and Subsequent Activation of Wnt/β-Catenin
Source: Oxid Med Cell Longev. 2022 Jan 6;2022:1084006. doi: 10.1155/2022/1084006 (PMC8758332; doi:10.1155/2022/1084006)
Supplement: Supplementary Materials — Figure S1: the sequences of primers and siRNAs used for qRT-PCR and siRNA cell transfections, respectively. Figure S2: hypoxia-triggered ROS did not affect cell viability. HaCaT cells were treated with hypoxia and NAC over a series of time points before analysis. (A) Levels of endogenous ROS were measured by a microplate reader. (B) Cell viability was determined by MTS assays. Mean ± SEM. n = 3. ∗∗∗P < 0.0001. Figure S3: KEGG enrichment analysis of upregulated mRNAs (A) and the enrichment of upregulated mRNAs in the Wnt pathway (B). Figure S4: Western blotting (A) and quantitative analysis (B) were employed to analyse the expression levels of HIF1 and SOX2 in hypoxia-treated HaCaT cells with or without ACF treatment for 12 h. Mean ± SEM. n = 3. ∗∗∗P < 0.0001. [file 1084006.f1.docx]

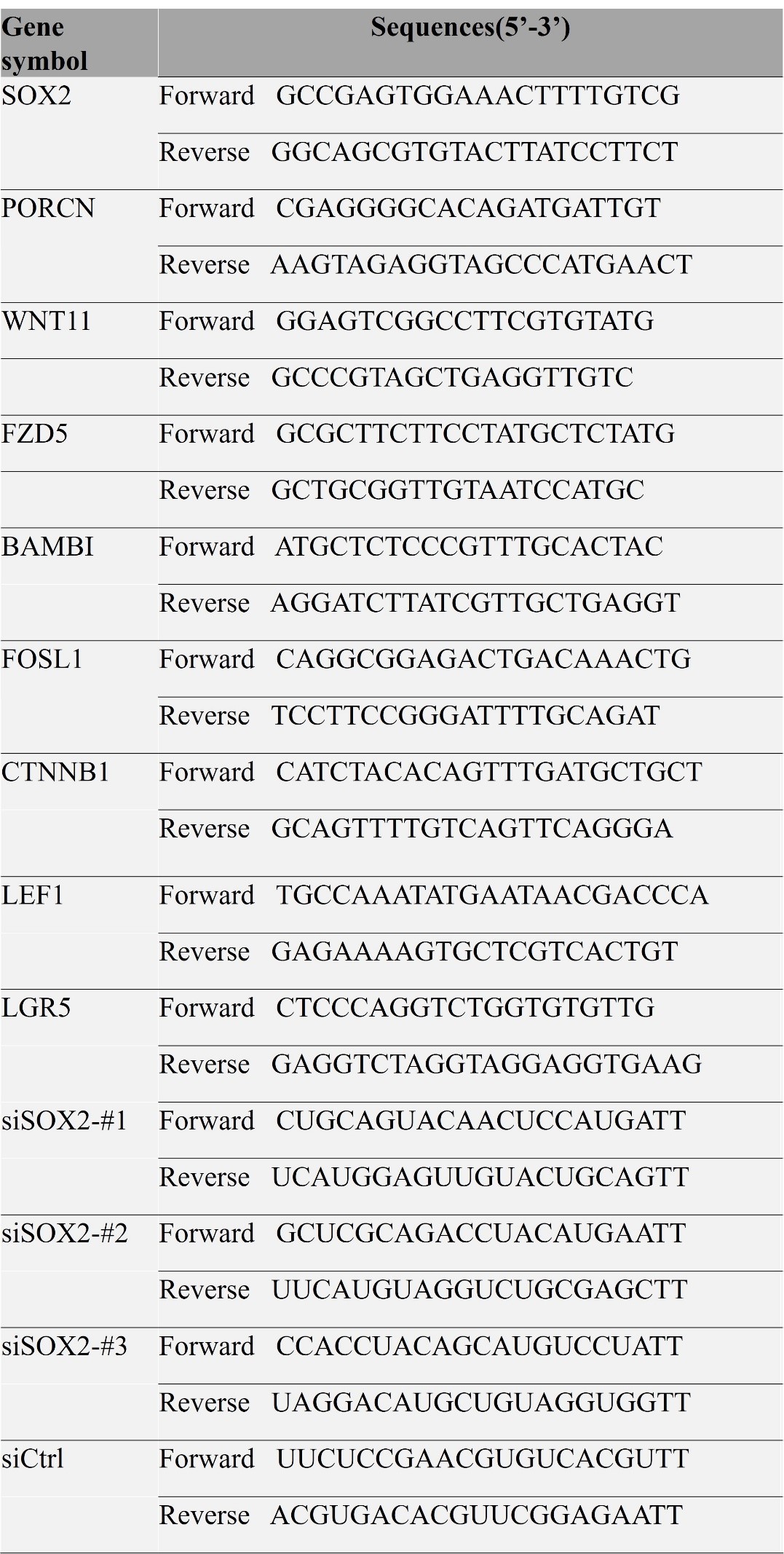


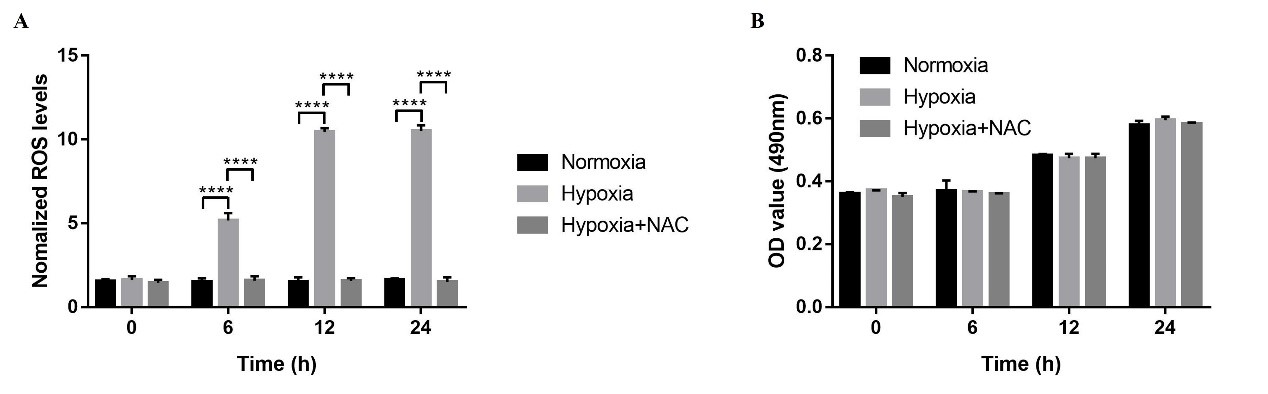


FIGURE S2. Hypoxia-triggered ROS did not affect cell viability. HaCaT cells were treated with hypoxia and NAC over a series of time points before analysis. **(A)** Levels of endogenous ROS were measured by a [microplate](#/javascript:;) [reader](#/javascript:;). **(B)** Cell viability was determined by MTS assays. Mean ± SEM. n = 3. ***P <0.0001.


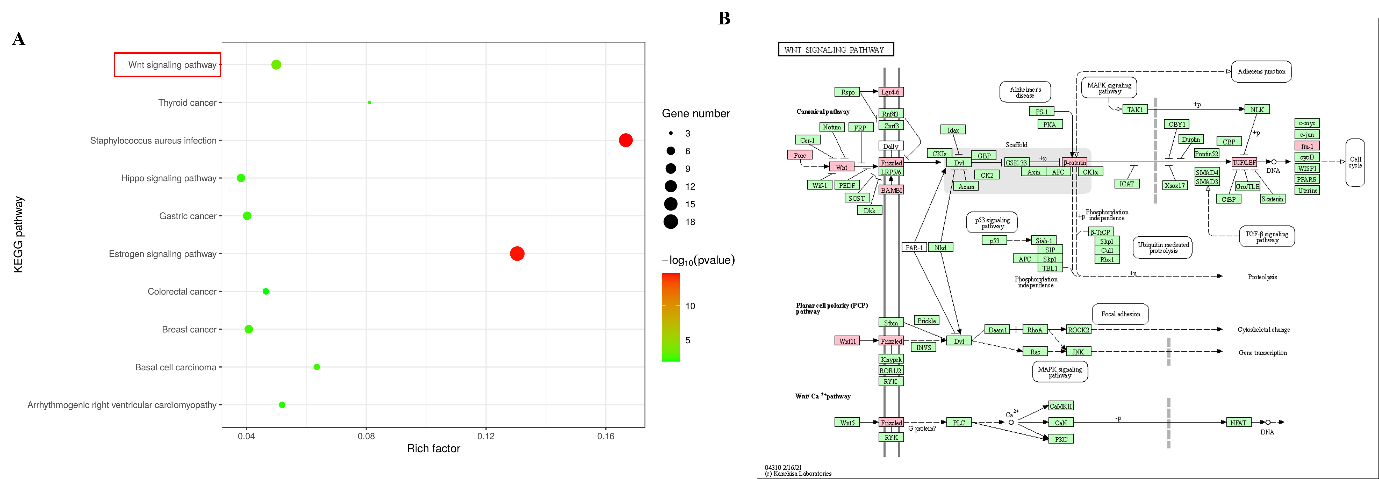


FIGURE S3. KEGG enrichment analysis of upregulated mRNAs **(A)** and the enrichment of upregulated mRNAs in the Wnt pathway **(B)**.


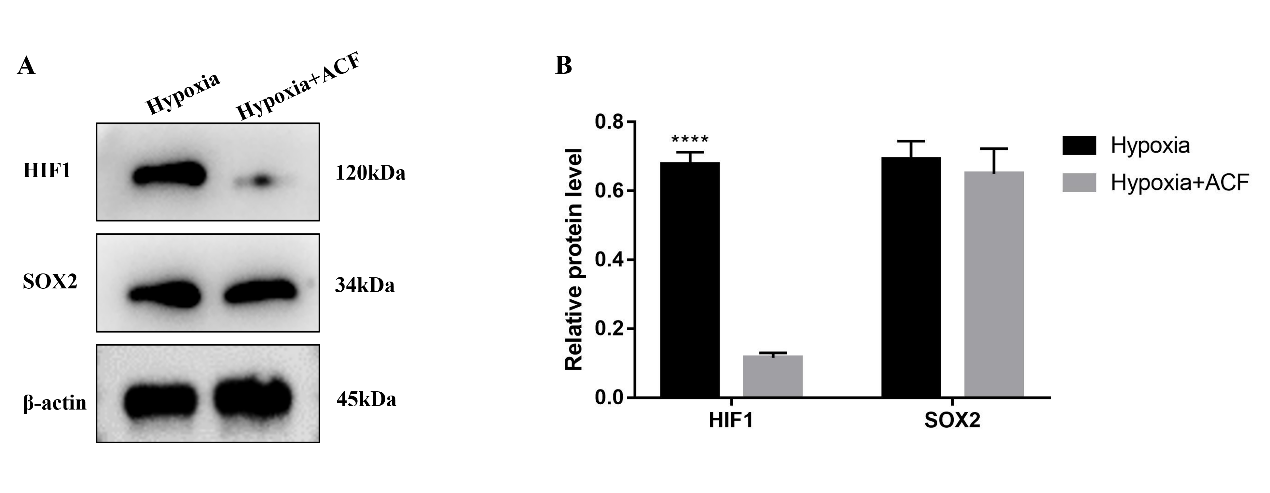


FIGURE S4. Western blotting **(A)** and quantitative analysis **(B)** were employed to analyse the expression levels of HIF1 and SOX2 in hypoxia-treated HaCaT cells with or without ACF treatment for 12 h. Mean ± SEM. n = 3. ***P <0.0001.W
